# Recurrent In-Stent Restenosis Treated With a Novel Technique: The Double-DCB Technique

**DOI:** 10.1016/j.jaccas.2026.107953

**Published:** 2026-04-24

**Authors:** An Shing Ang, Thet Khaing, Bharat Khialani, Kwok Kong Jason Loh, Hee Hwa Ho

**Affiliations:** a Department of Cardiology, Woodlands Hospital, Singapore; b Department of Cardiology, Tan Tock Seng Hospital, Singapore

**Keywords:** drug-coated balloons, percutaneous coronary intervention, stents

## Abstract

Percutaneous coronary intervention plays an important role in the management of both acute and chronic coronary syndrome. Despite advances in stent technology, in-stent restenosis remains a major Achilles heel in percutaneous coronary intervention and can confer high risk of adverse outcomes for patients. We describe a novel technique (the double-DCB technique) for the treatment of recurrent in-stent restenosis by applying 2 different drug-coated balloons, thereby avoiding the implantation of another layer of metal in our patients. More studies are required to understand the long-term outcomes of this technique and compare it with other treatment modalities in a randomized setting.

## Visual Summary

**Figure:**
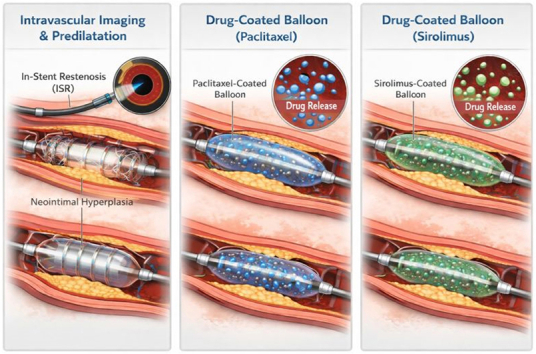

**Visual Summary:**
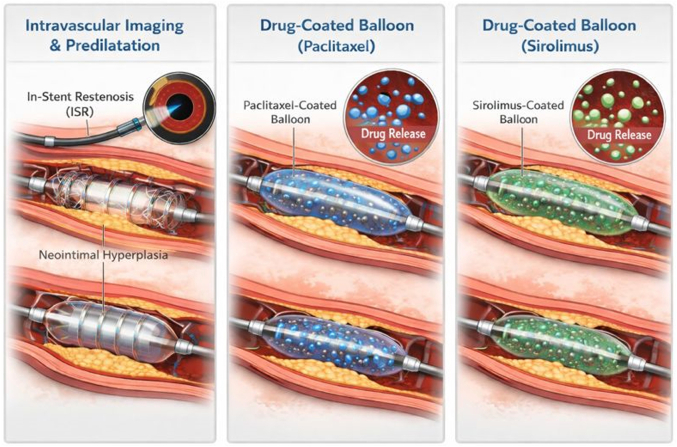
Double-DCB Technique for the Treatment of Recurrent In-Stent Restenosis First, intravascular imaging should be performed to exclude any mechanical causes of in-stent restenosis. After adequate lesion preparation, the paclitaxel DCB is first deployed, followed by the sirolimus DCB. DCB = drug-coated balloon.

Percutaneous coronary intervention (PCI) plays an important role in the management of both acute and chronic coronary syndrome. In the past 2 decades, the use of metallic stents has become widespread as more patients undergo PCI. While advances in stent technology (from bare-metal stents to drug-eluting stents [DESs]) have reduced the incidence of in-stent restenosis (ISR) from 30% at 6 months to 5% to 15% after 5 years with DESs, there still exists a significant number of patients with ISR.^1^

PCI for patients with ISR confers a higher risk of mortality, myocardial infarction, target-vessel revascularization, and stent thrombosis.^1^ For treatment of bare-metal stent ISR, drug-coated balloons (DCBs) have shown similar long-term safety and efficacy when compared to DESs.^2^ While the latest evidence favors treating ISR in DES with another layer of stent,^2^ it has been shown that having more layers of metal portends a poorer long-term prognosis for patients.^3^

In this case series, we describe a novel combination therapy for the management of recurrent ISR using sequential overlapping DCBs, one paclitaxel coated and one sirolimus coated. By targeting distinct pathways involved in neointimal proliferation, this dual-drug approach may provide a synergistic antiproliferative effect. Importantly, the technique—termed the double-DCB technique—enables effective lesion treatment while avoiding the implantation of an additional stent layer. To our knowledge, based on a comprehensive review of the existing literature, this is the first case series to describe the use of sequential overlapping paclitaxel- and sirolimus-coated DCBs for the treatment of recurrent ISR.

## Methodology

The double-DCB technique involves 3 steps. 1) Intravascular imaging is performed to exclude any mechanical causes of stent failure and to assess the mechanism of ISR. 2) Meticulous lesion preparation is undertaken with specialty scoring or cutting balloons, with adjunctive calcium modification when indicated to ensure optimal luminal expansion. Once adequate lesion preparation is achieved,^4^ 3) sequential drug delivery is performed: a paclitaxel DCB is deployed first, followed by a sirolimus DCB. The balloons are sized 1:1 with the vessel diameter and are inflated for at least 30 seconds each.

The order of deployment is intentional. The paclitaxel DCB is deployed first, as paclitaxel is more lipophilic and taken up by the cells quickly, making it less susceptible to disruption by subsequent balloon inflation. In contrast, sirolimus is less lipophilic and requires additional reservoirs to facilitate drug transfer to the vessel wall. We use the Selution SLR DCB (Cordis), which uses proprietary micro-reservoir technology to deliver sirolimus into the vessel walls. This technology allows a controlled and sustained release of the drug, maintaining therapeutic levels for over 90 days. Moreover, the Cell Adherent Technology of the Selution SLR contains and protects the micro-reservoir during inflation of the DCB. Deploying the paclitaxel DCB first therefore minimizes the risk of disrupting the sirolimus reservoirs and preserves effective drug delivery.

## Case Presentations

### Case 1

Patient 1 was a 62-year-old man with cardiovascular risk factors of hypertension and tobacco use. He first presented with a non–ST-segment elevation myocardial infarction (NSTEMI) in February 2022 and underwent intravascular ultrasound (IVUS)–guided PCI to the ostial to mid right coronary artery (RCA) with a 3.5 × 48 mm DES.

**Figure 1:**
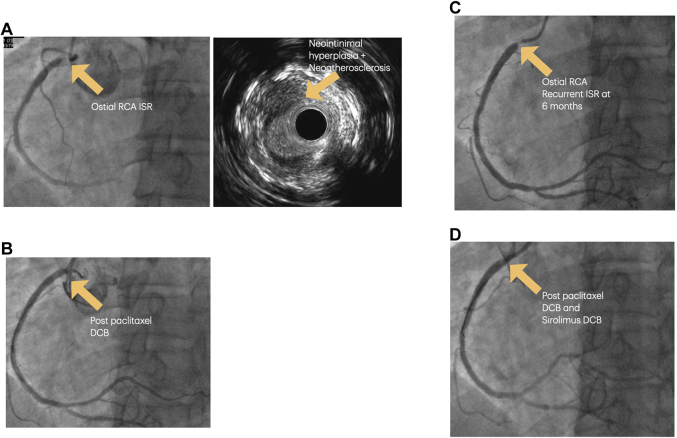
Preprocedural and Postprocedural Imaging for Case 1 (A) Severe ostial RCA ISR, secondary to neointimal atherosclerosis (B) Angiographic result after paclitaxel DCB only. (C) Recurrent ostial RCA ISR at 6 months. (D) Angiographic result after paclitaxel DCB and sirolimus DCB. DCB = drug-coated balloon; ISR = in-stent restenosis; RCA = right coronary artery.

He presented with angina in October 2022. Coronary angiogram revealed a focal 95% ISR in the ostial RCA stent (Figure 1A). IVUS showed that the mechanism of ISR was neoatherosclerosis, with no mechanical failure of the stents. After lesion modification with a scoring balloon, the ISR was treated with a 4.0 × 20 mm paclitaxel DCB (Figure 1B).

He re-presented with another episode of NSTEMI <6 months later. Coronary angiogram revealed a recurrent ISR at the ostial RCA (Figure 1C). IVUS showed that the mechanism of ISR was neointimal hyperplasia, with no mechanical failure of the stents. After adequate lesion preparation with a 3.5-mm scoring balloon at high pressures, we performed our double-DCB technique, first deploying a 4.0 × 20 mm paclitaxel DCB followed by a 4.5 × 20 mm sirolimus DCB (Figure 1D) for 60 seconds each, sizing our DCB 1:1 with the vessel diameter as much as possible.

The patient has undergone followed-up in the outpatient clinic over the past 2 years and has remained symptom free.

### Case 2

Patient 2 was a 64-year-old woman with cardiovascular risk factors of hypertension, hyperlipidemia, and mixed connective tissue disease on azathioprine and prednisolone. She also has moderate calcific aortic stenosis. She first presented with an inferior ST-segment elevation myocardial infarction (STEMI) in May 2022 and underwent IVUS-guided and intravascular lithotripsy–assisted PCI with a 3.5 × 32 mm DES.

**Figure 2:**
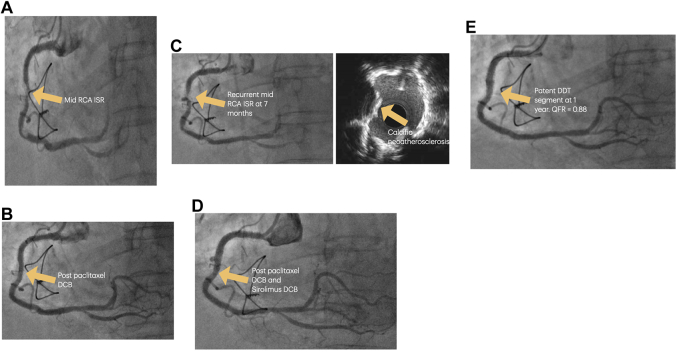
Preprocedural and Postprocedural Imaging for Case 2 (A) Severe mid-RCA ISR. (B) Angiographic result after paclitaxel DCB only. (C) Recurrent ostial RCA ISR at 7 months, secondary to calcific neoatherosclerosis. (D) Angiographic result after paclitaxel DCB and sirolimus DCB. (E) Angiographic result at 1 year after the double-DCB technique (DDT), with nonsignificant quantitative flow ratio (QFR: 0.88). Abbreviations as in Figure 1.

She presented with angina in November 2022. Coronary angiogram showed a focal mid-RCA ISR (Figure 2A). Lesion preparation was performed with a scoring balloon before the ISR was treated with a 3.5 × 30 mm paclitaxel DCB (Figure 2B).

She re-presented with angina in June 2023. Coronary angiogram showed a recurrent mid-RCA ISR (Figure 2C). IVUS indicated the mechanism of ISR was calcific neoatherosclerosis, with no mechanical failure of the stent (Figure 2C). After adequate lesion preparation was performed with a 3.5-mm intravascular lithotripsy balloon followed by a 3.5-mm scoring balloon at high pressures, we performed our double-DCB technique, first deploying a 4.0 × 30 mm paclitaxel DCB followed by a 4.0 × 20 mm sirolimus DCB (Figure 2D) for 60 seconds each, sized 1:1 with the vessel diameter.

One year later, the patient again re-presented with angina. A coronary angiogram was performed and showed only mild stenosis in the segment treated with our double-DCB technique (Figure 2E). A quantitative flow ratio analysis of the RCA was performed, which was nonsignificant at 0.88. Transthoracic echocardiography showed progression of her aortic stenosis to severe, and she subsequently underwent transfemoral aortic valve replacement and has since remained symptom free.

### Case 3

Patient 3 was a 75-year-old man with cardiovascular risk factors of hypertension, smoking, and diabetes mellitus. He first presented with unstable angina pectoris in June 2023 and underwent PCI to both the left anterior descending artery (LAD) and RCA. No details of the PCI were available, as it was performed at a private institution.

**Figure 3:**
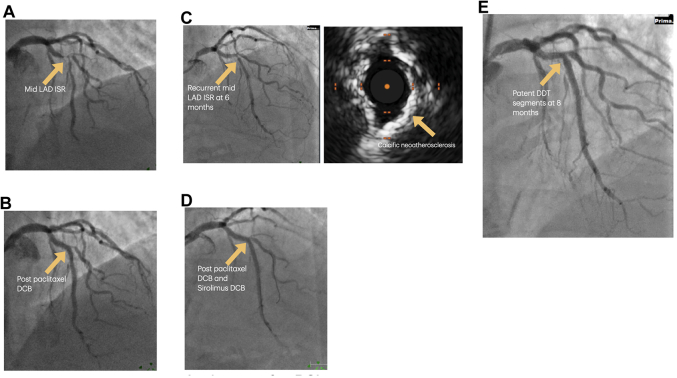
Preprocedural and Postprocedural Imaging for Case 3 (A) Severe mid-LAD ISR. (B) Angiographic result after paclitaxel DCB only. (C) Recurrent mid LAD ISR at 6 months, secondary to calcific neoatherosclerosis and neointimal hyperplasia. (D) Angiographic result after paclitaxel DCB and sirolimus DCB. (E) Angiographic result at 8 months after the double-DCB technique (DDT). DCB = drug-coated balloon; ISR = in-stent restenosis; LAD = left anterior descending artery.

He re-presented with angina in February 2024. Coronary angiogram showed a severe mid-LAD ISR (Figure 3A), which was prepared with a scoring balloon before the ISR was treated with a 3.0 × 15 mm paclitaxel DCB (Figure 3B).

He again re-presented with angina in August 2024. Coronary angiogram showed a recurrent LAD ISR (Figure 3C) and RCA chronic total occlusion ISR. IVUS of the LAD showed that the mechanism of ISR was calcific neoatherosclerosis and neointimal hyperplasia in the LAD on a background of eccentrically expanded stents (Figure 3C). The lesion was modified with 3.0-mm scoring and 3.25-mm noncompliant balloons at high pressures to modify the plaque and improve stent expansion. We achieved good balloon expansion on orthogonal views before we performed our double-DCB technique, first deploying a 3.5 × 25 mm paclitaxel DCB followed by a 3.5 × 25 mm sirolimus DCB in the proximal vessel, and a 3.0 × 20 mm paclitaxel DCB followed by a 3.0 × 20 mm sirolimus DCB in the distal vessel for 60 seconds each, sized 1:1 with the vessel diameter (Figure 3E).

The patient was brought back for PCI to the RCA, as he had persistent symptoms (albeit improved) 8 months later. A relook angiogram of the LAD showed minor restenosis within the segment treated with our double-DCB technique (Figure 3F). He has remained symptom free after PCI to the RCA.

## Discussion

ISR continues to remain as a major Achilles heel in PCI despite advances in PCI technology. Patients with ISR are at higher risk of recurrent major ischemic events.^1^ A previous multicenter registry reviewing more than 48,000 de novo lesions across 15 years demonstrated significant rates of recurrence of ISR after first, second, and third reintervention at 8.3%, 17.1%, and 22.8%, respectively.^5^

The pathogenesis of ISR comprises a biological mechanism that involves neointimal hyperplasia and neoatherosclerosis, as well as a mechanical component, which stems from stent underexpansion associated with undersizing, calcification, or stent fracture.^1^

DCBs represent an attractive strategy to manage ISR patients by delivering an antiproliferative drug without the addition of a scaffolding layer. It gives the primary physician more maneuverability when tailoring the antiplatelet regimes of patients with high bleeding risk. Moreover, treatment of ISR with DCBs is already given a Class I indication based on the 2018 European Society of Cardiology/European Association for Cardio-Thoracic Surgery myocardial revascularization guidelines. A previous study reflects this sentiment among operators in the Asia-Pacific region, where the majority would still opt for DCB over a DES strategy when treating patients with ISR.^6^ With a contemporary study^2^ favoring DESs over DCBs in the treatment of DES-ISR, there might be a paradigm shift away from DCBs. However, multiple stent insertions are associated with a higher risk of restenosis,^3^ suggesting that each additional layer of metal adds additional risk of restenosis and hence poorer clinical outcomes.

We believe that when treating a patient with recurrent ISR, intravascular imaging should be performed to elucidate the underlying cause of the ISR. If the underlying cause is mechanical, it should be treated with a mechanical solution (eg, deploying another layer of stent for stent fracture, or expanding an underexpanded stent). However, if the underlying process is biological, we propose considering our double-DCB technique, combining paclitaxel with sirolimus to create a synergistic antiproliferative effect to better counteract the biological processes driving ISR, once adequate lesion preparation and calcium modification as required have been performed. We hypothesize that by inhibiting intimal proliferation in this 2-pronged approach, the progression of ISR can be retarded more effectively.

There may be a theoretical risk that exposure to paclitaxel and sirolimus may cause endothelial injury owing to their mechanism of action. However, the dual application of paclitaxel and sirolimus has been researched in an animal (rabbit and porcine) study, with promising results.^7^ In the rabbit model, DCBs coated with paclitaxel and sirolimus showed more potent inhibition of intimal proliferation compared with paclitaxel-only DCB in the acute phase, and there was also evidence of better healing of medial injury in the subacute phase in the porcine model.^7^ In our 3 patients, we managed to avoid implanting another layer of stent, which may have been detrimental to our patients in the long term.

There is a wealth of literature demonstrating the efficacy of paclitaxel DCBs, and literature demonstrating the clinical efficacy of Selution SLR is growing. Selution SLR has been shown to be a safe and feasible treatment option in patients undergoing primary PCI; preliminary data from a study by Ang et al^8^ demonstrated a 100% procedural success rate and low rates of adverse events at 1 year in patients with complex lesions treated with Selution SLR. More recently, the SELUTION DeNovo Trial,^9^ an all-comers randomized controlled trial with 3,326 patients, demonstrated noninferiority at 1 year of Selution SLR compared with DES, which is still widely considered the treatment of choice for the treatment of de novo lesions. The SELUTION4ISR trial^10^ also demonstrated that the Selution SLR was noninferior to the current standard of care for treatment of ISR at 1 year.

Our 3 cases demonstrated good outcomes in our patients treated with the double-DCB technique for at least 1 year, suggesting that it may be a safe and feasible treatment of choice in patients with recurrent ISR if a mechanical cause is excluded. Beyond application of 2 different medications on the vessel wall, adequate lesion preparation is a key step in the double-DCB technique. However, given the limitations of a case series, this technique may not be generalizable to the wider population yet. More studies are required to evaluate the long-term outcomes and safety data of this treatment technique and to compare it in a randomized controlled trial against contemporary treatments. In our case series, we did not discuss other treatment modalities that could have been complementary, such as brachytherapy, as it is not available in our country.

## Conclusions

In this case series, we describe a novel combination therapy involving 2 different DCBs—the double-DCB technique—in the treatment of recurrent ISRs. Application of both paclitaxel and sirolimus DCBs allows for a synergistic inhibition of intimal proliferation. This potentially reduces the need for more layers of metallic stents, which may be detrimental for patients. This case series is hypothesis-generating, and more data and studies are required to understand the long-term outcomes of this technique and compare it to other treatment modalities in a randomized setting.

## Funding Support and Author Disclosures

The authors have reported that they have no relationships relevant to the contents of this paper to disclose.

**Take-Home Messages**

• Intravascular imaging is important to assess the mechanisms of in-stent restenosis.
• After excluding any mechanical causes of stent failure, adequate lesion preparation to achieve adequate luminal gain is important before drug-coated balloon angioplasty.
• Application of the double-DCB technique may reduce the need for additional layers of stents in patients with recurrent in-stent restenosis after excluding mechanical causes of stent failure.
